# Using multi-step proposal distribution for improved MCMC convergence in Bayesian network structure learning

**DOI:** 10.1186/s13637-015-0024-7

**Published:** 2015-06-20

**Authors:** Antti Larjo, Harri Lähdesmäki

**Affiliations:** 1 Department of Information and Computer Science, Aalto University, FI-00076Aalto, Finland; 2 Department of Signal Processing, Tampere University of Technology, Tampere, FI-33101 Finland; 3 Turku Centre for Biotechnology, Turku University, Turku, FI-20521 Finland

**Keywords:** Bayesian network, Structure learning, MCMC, Proposal distribution

## Abstract

Bayesian networks have become popular for modeling probabilistic relationships between entities. As their structure can also be given a causal interpretation about the studied system, they can be used to learn, for example, regulatory relationships of genes or proteins in biological networks and pathways. Inference of the Bayesian network structure is complicated by the size of the model structure space, necessitating the use of optimization methods or sampling techniques, such Markov Chain Monte Carlo (MCMC) methods. However, convergence of MCMC chains is in many cases slow and can become even a harder issue as the dataset size grows. We show here how to improve convergence in the Bayesian network structure space by using an adjustable proposal distribution with the possibility to propose a wide range of steps in the structure space, and demonstrate improved network structure inference by analyzing phosphoprotein data from the human primary T cell signaling network.

## Introduction

Probabilistic graphical models are a class of models often used in various application fields. Their popularity is partly due to their appealing visual representation of the model structure that in many cases is also capturing the real structure of the underlying system. Bayesian networks (BNs) are probabilistic graphical models that have received broad attention in biological sciences, e.g., in gene regulatory and signaling network modeling [1–5]. BNs are also utilized, for example, in medical diagnostics [6], speech recognition [7], reliability and risk analysis [8], and numerous other probabilistic decision making applications [9]. BNs are able to incorporate prior information as well as model the dependency structure of a multivariate joint probability distribution. This structure is often likened to the real network structure by interpreting the model as causal [10]. Correspondingly, in many applications, a key question is the inference of the underlying network structure from experimental data.

Factors complicating BN structure learning include superexponentially growing structure-space size as the number of nodes increases. This prohibits exhaustive evaluation for most practical applications and instead forces to utilize heuristic search techniques, such as hill climbing, which can suffer from finding mostly only local maxima, or more preferably sophisticated sampling methods, like Markov Chain Monte Carlo (MCMC) methods.

MCMC methods present their own problems, among which a major one is the speed of convergence. Convergence is influenced by the sheer size of the space of possible structures and the shape of the posterior landscape, which can contain local maxima that are hard for the chain to escape. Size of the search space can be limited, e.g., by enforcing criteria like maximum in-degree, but slow convergence and local minima can still remain a bottleneck for MCMC.

To improve MCMC structure inference methods, various types of efficient methods have been proposed. For example, [11] proposed devising proposal distributions with an edge reversal technique. Notably, one can also cast structure learning into a problem of learning “superstructures” (sets of structures), like trying to identify the order of nodes [12], and improvements upon it [13, 14]. Other notable improvements in BN structure learning also include the work in [15], where dynamic programming is utilized to calculate the posterior probabilities of all BNs in exponential time, and variations of this [16]. Although the dynamic programming approach can identify the optimal network structure exactly, it is applicable only to networks with limited number of variables. In addition, the methods based on dynamic programming or node orders make it more challenging to use arbitrary informative structural prior distributions.

The Bayesian network structure is restricted to directed acyclic graph (DAG). MCMC in the space of DAGs is more challenging than in continuous space because, e.g., the exceedingly large discrete search space and the acyclicity constraint, which make the search-space exploration computationally demanding and all but the simplest proposal distributions more difficult to define. Also, due to the latter reason, adaptive MCMC methods have been difficult to implement in DAG space.

The MCMC strategy presented here uses an adjustable proposal distribution and improves convergence of the MCMC chains practically without increasing the computational costs. Indeed, the proposed method often decreases computational load by enabling the chains to escape peaks of local maxima much more efficiently.

## Background

### Bayesian networks

A Bayesian network (BN) [17] is a (semi-graphical) representation of a joint probability distribution, describing also the dependencies between variables (dependency structure). Formally, given a set of random variables $\mathcal {X}=\{X_{1},..., X_{n}\}$, a Bayesian network is defined as a pair (*G*,**θ**), where *G* is a directed acyclic graph (DAG) whose *n* nodes represent the variables in $\mathcal {X}$ and edges give a graphical representation of the conditional independencies between these variables so that each node *X*_*i*_ is conditionally independent of its nondescendants given its parents in *G*. Parameter set **θ** defines the conditional probability distributions of these variables. *G* gives the factorization of the joint distribution over $\mathcal {X}$ as

(1) $$ P\left(X_{1},..., X_{n}|G,\mathbf{\theta}\right) = \prod_{i=1}^{n}{P\left(X_{i}|\text{Pa}_{G}(X_{i}),\mathbf{\theta}_{i}\right)},  $$

where Pa_*G*_(*X*_*i*_) is the set of parents of node *X*_*i*_ in *G*, and **θ**_*i*_ denotes the parameters for the distribution of *X*_*i*_ conditional on its parents. Thus, BNs can be used to model probability distributions that respect the directed factorization property, i.e., the distribution factorizes according to the DAG.

In searching for the structure that most probably generated the data, of main interest is the posterior probability of a DAG *G* given the data *D*

(2) $$  P\left(G|D\right) = \frac{P\left(D|G\right)P(G)}{P(D)},  $$

where *P*(*G*) is the prior probability of *G*, $P(D)=\sum _{G' \in \mathcal {G}_{n}}{P\left (D|G'\right)P(G')}$ is the probability of data, $\mathcal {G}_{n}$ is the set of all possible DAG structures with *n* nodes, and

(3) $$  P\left(D|G\right) = \int_{\theta}{P\left(D|G,\mathbf{\theta}\right)P\left(\mathbf{\theta}|G\right) d\theta}  $$

is the marginal likelihood.

For certain choices of probability distributions and parameter priors, it is possible to arrive at a closed form solution for the marginal likelihood. The two main cases are multinomial distributions with (independent) Dirichlet priors [18, 19] and Gaussian distributions with normal-Wishart priors [20] (called BDe and BGe models in [19], respectively). Here, we will focus on discrete-valued data and BNs having multinomial conditional probability distributions although the proposed structure MCMC is applicable for any distribution.

### Structure MCMC for Bayesian networks

Ideally, we would like to have the whole posterior distribution of DAGs and calculate our further analyses based on that. But since the number of different DAGs grows superexponentially with *n*, evaluating any score for all possible structures is prohibitive for all but the smallest of *n* (*n*≤6 or so). Thus, one is forced to sample the posterior distribution with a method like MCMC, as is done in this study. Also, it is often not justified to take just a single DAG from the posterior due to, for example, a small dataset making the posterior spread, or multimodality of the posterior. Instead, to better represent the posterior, it is sensible to take a set of network structures which have a high posterior probability.

In the following, we shortly review the basic MCMC for BN structure learning as well as some convergence diagnostics.

#### Structure MCMC

In order to sample from the posterior distribution of structures, a Markov chain is set up so that its target distribution is *P*(*G*|*D*) [21]. This is done using the Metropolis-Hastings algorithm which consists of proposing a move from structure *G* to *G* ^′^ with probability *Q*(*G* ^′^|*G*) and accepting the move with probability

(4) $$ \min\left\{ 1, \frac{P(D|G')P(G')Q(G|G')}{P(D|G)P(G)Q(G'|G)} \right\}.   $$

This action is called a Metropolis-Hastings (MH) step/move. The probability distribution *Q*() is called proposal distribution (or sometimes jumping distribution), and the ratio $\frac {Q(G|G')}{Q(G'|G)}$ is called the Hastings ratio. The proposal distribution in BN structure learning is most often defined as

(5) $$ Q(G'|G)=\left\{ \begin{array}{ll} \frac{1}{|\mathcal{N}_{Q}(G)|}, & \text{if} G' \in \mathcal{N}_{Q}(G) \\ 0, & \text{if} G' \notin \mathcal{N}_{Q}(G) \end{array} \right.   $$

where $\mathcal {N}_{Q}(G)$ is the neighborhood of *G* reachable by *Q*(·|*G*), being most often the set of DAGs that are the result of a single edge modification (addition, deletion, reversal) to *G*, and $|\mathcal {N}_{Q}(G)|$ is the cardinality of this set.

While in some applications the proposal distribution can be symmetric (i.e., *Q*(*G*|*G* ^′^)=*Q*(*G* ^′^|*G*)) and thus the Hastings ratio unity (in which case the Metropolis-Hastings algorithm is called simply Metropolis algorithm), in the context of Bayesian network structures and (5), it is generally not, which is due to the acyclicity requirements and thus varying neighborhood sizes. This fact complicates the process of making new proposal distributions and is one of the main reasons for generally using only simple (one-step) proposal distributions, although approximating the Hastings ratio to be unity can also be considered.

#### Convergence

After running the chain long enough (burn-in phase), it should have attained its stationary distribution which corresponds to *P*(*G*|*D*). Then, by taking a large enough sample from the chain, we get a good estimate for this true posterior distribution. The problem is in knowing whether a chain has converged to the true stationary distribution or not. Several convergence assessment methods have been proposed for MCMC in continuous space [22] but not many suitable for BN structure learning.

One frequently used indicator for convergence is the similarity of edge posterior probabilities calculated from two or more independent chains. Posterior probability of a feature *f* (e.g., an edge) can be calculated for a sample from an MCMC chain as [1]

(6) $$  P\left(f|D\right)=\sum\limits_{G\in {\mathcal {G}}_{n}}P(G|D)I_{f}(G)\approx\frac{1}{|\mathcal{G}_{S}|}\sum\limits_{G\in \mathcal{G}_{S}}I_{f}(G),  $$

where *I*_*f*_ is an indicator function, i.e., *I*_*f*_(*G*)=1 if graph *G* contains the wanted feature and *I*_*f*_(*G*)=0 otherwise; $\mathcal {G}_{S}$ is the set of sampled graphs; and $|\mathcal {G}_{S}|$ is the number of sampled graphs. Noticeable deviations of edge posterior probabilities between independent chains then indicate that the chains have not converged to the same stationary distribution.

In addition to the above convergence diagnostic, in this study, we use score plots (or trace plots), which are simply the score (or the likelihood in (3) when given uniform priors like here) plotted for each of the sampled DAGs. Similar distribution of scores from independent chains suggests convergence. Investigating score plots can usually show easily which chains are stuck on areas with lower scores than some other chains.

These are only necessary (not sufficient) conditions for convergence, and in fact, there is generally no way to assure that a chain is converged to the target distribution and that a finite sample from it is sufficiently representative of the true target distribution [22].

## Proposal distribution

In the case of the standard MCMC, the only tunable parameter of the sampling process, besides burn-in and sample sizes, is the proposal distribution. Needed sample sizes are dependent on the dataset size, data properties (i.e., whether the model search space with this dataset is multimodal or not), and also properties of the proposal distribution.

To give some rationale for tweaking the proposal distribution, consider the following: A correctly set-up MCMC chain is guaranteed to converge to the target distribution, given that the burn-in phase is long enough. As the DAG structure space grows (super)exponentially with respect to the number of nodes in a network, the required run times for MCMC chains grow also. This is at least partly due to the fact that as the size of the search space grows, it is also more possible for the chain to get stuck on local maxima for a long time. Some help for this can be found by finding alternative proposal distributions for the MH moves.

There is also the risk of false estimation if the convergence is very slow, as a chain may spend too much time in a locally high-scoring region. More specifically, if a region or area of the search space (or structure/DAG space) consists of a set of neighboring DAGs with relatively equal scores, and this set is being surrounded by a set of considerably lower scoring DAGs, then the probability of escaping such region can be very low, and if the target distribution is estimated from such chain, it can be badly skewed (see, e.g., [23]) and even pass a chosen convergence criterion. Ideally, a proposal distribution should be one that is capable of moving both within and between areas of high scoring structures, allowing the sample to include DAGs from several of them, while not getting stuck on such areas for too long.

The usual choice of proposal distribution in the context of learning Bayesian network structures is to consider single edge changes to the DAG: addition, deletion, or reversal of an edge. Such a proposal distribution is called single-step or one-step in this paper. This reflects the ability to make one step to the *neighborhood* of a DAG, defined as the set of DAGs for which there is a probability greater than zero of being proposed by the proposal distribution in use, i.e., the neighborhood of *G* is defined as {*G* ^′^|*Q*(*G* ^′^|*G*)>0}.

Aside from classical single-edge proposal distributions, other proposal distributions have also been presented. These include inclusion order [24], optimal reinsertion operator [25], and edge reversal moves [11]. Searching in the space of equivalence classes has also been suggested [26]. Also [27] discusses larger than one-step neighborhoods in the context of Gibbs sampling.

When constructing the proposal distribution, the acyclicity of the proposed structures must be taken into account, for which an efficient algorithm has been proposed [28]. However, the main difficulty in constructing neighborhoods larger than single edge is that their size grows superexponentially and the acyclicity checks start to get computationally very demanding due to the need to calculate the Hastings ratio in (4), for which the sizes of the neighborhoods are needed (in case of the usual uniform proposal distribution). The sizes of these neighborhoods (and thus also the time required for acyclicity checks) grow exponentially with the step length.

Below, we show that in many cases the neighborhood sizes need not be evaluated, thus bypassing this problem of growing neighborhood sizes.

### DAG space

Taking a fixed number of nodes in $\mathcal {X}$, the space of possible model structures (here DAGs) can be described with an undirected graph where each node stands for one structure and edges between them denote possible transitions between these. The most elementary transitions consist of single-edge modifications (addition, deletion, reversal) to the structure that do not introduce cycles. The edges are undirected since for each transition consisting of a single-edge modification, there is a reverse transition with the reverse edge modification (i.e., addition ⇔ deletion or reversal ⇔ reversal), and thus, if there exists a transition from *G*_*i*_ to *G*_*j*_, then there necessarily is also a transition from *G*_*j*_ to *G*_*i*_.

A transition of length *t* between two structures *G*_*i*_ and *G*_*k*_ is a walk of length *t* between their respective nodes in the model structure space, i.e., $\phantom {\dot {i}\!}r = (G_{i}, G_{r_{1}}, G_{r_{2}},..., G_{r_{t-1}}, G_{k})$. Note that this is not necessarily a simple path, i.e., the same vertices can appear more than once in the walk. Also note that due to the undirectedness of the graph, each transition can be traversed in both directions, and therefore, for each *r*, there is a reverse transition *r* ^′^, for example, $\phantom {\dot {i}\!}r' = (G_{k}, G_{r_{t-1}}, G_{r_{t-2}},...,G_{r_{1}}, G_{i})$ in the above case.

### Multi-step proposal distributions

Let *Q* ^*t*^(*k*|*i*,**r**) be a proposal distribution from structure *i* to *k* that can be decomposed into *t* (here *t*>1) independent (sub)distributions *Q*_*j*_, *j*=1,…,*t*, and **r**=(*r*_1_,*r*_2_,…,*r*_*t*−1_) is a tuple of intermediate structures so that the whole move is *i*→*r*_1_→⋯→*r*_*t*−1_→*k*. Note that we use here only the indices of structures (e.g., *i* to denote *G*_*i*_) to keep the notation more readable. The probability of proposing the move is then

(7) $$\begin{array}{@{}rcl@{}} Q^{t}\left(k|i,\mathbf{r}\right) &=& Q_{1}\left(r_{1}|i\right) Q_{2}\left(r_{2}|r_{1}\right) \cdots Q_{t}\left(k|r_{t-1}\right)\\ &=& Q_{1}\left(r_{1}|i\right)Q_{t}\left(k|r_{t-1}\right)\prod\limits_{j=2}^{t-1}{Q_{j}\left(r_{j}|r_{j-1}\right)}, \end{array} $$

where each subdistribution *Q*_*j*_(*r*_*j*_|*r*_*j*−1_) can be any function giving a probability for the move *r*_*j*−1_→*r*_*j*_.

When using the proposal distribution *Q* ^*t*^(*k*|*i*,**r**), the move from *i* to *k* is in general possible using several different **r**. For example, if *k* is otherwise the same structure as *i* but with two edges *a* and *b* added, it is possible to add first either *a* or *b* and after that the other. We note the set containing all such possible routes as $R_{i\rightarrow k}^{Q^{t}}$. The probability that *Q* ^*t*^(·|*i*) proposes a move from *i* to *k* is then

(8) $$ Q^{t}\left(k|i\right) = \sum\limits_{\mathbf{r}\in R_{i\rightarrow k}^{Q^{t}}} Q^{t}\left(k|i,\mathbf{r}\right).  $$

#### Extending standard single-step proposal distribution

We consider extending the usual proposal distribution consisting of one single-edge modification to a multi-step proposal distribution, capable of proposing transitions of length *t* so that it consist of *t* sequential single-edge modifications, each drawn from a uniform distribution of the corresponding neighborhood. In this case, each of the subdistributions is defined as $Q\left (r_{i+1}|r_{i}\right) = \frac {1}{q(r_{i})}$, where *r*_*i*_ and *r*_*i*+1_ are DAGs differing by a single-edge modification and *q*(·) is a function giving the number of neighboring structures for a DAG. The neighborhood size can be calculated by evaluating the number of all possible single-edge modifications to the current DAG yielding an acyclic graph.

Now the probability of proposing a transition of length *t* from *G*_*i*_ to *G*_*k*_ is

(9) $$\begin{array}{@{}rcl@{}} Q^{t}\left(k|i\right) &=& \sum\limits_{\mathbf{r}\in R_{i\rightarrow k}^{Q^{t}}}{\frac{1}{q(i)}\prod\limits_{j=1}^{t-1}{\frac{1}{q(r_{j})}}}\\ &=& \frac{1}{q(i)}\sum\limits_{\mathbf{r}\in R_{i\rightarrow k}^{t}}{\prod\limits_{j=1}^{t-1}{\frac{1}{q(r_{j})}}},  \end{array} $$

and the probability of proposing a transition back from *G*_*k*_ to *G*_*i*_ is similarly

(10) $$\begin{array}{@{}rcl@{}} Q^{t}\left(i|k\right) &=& \frac{1}{q(k)}\sum\limits_{\mathbf{r}'\in R_{k\rightarrow i}^{t}}{\prod\limits_{j=1}^{t-1}{\frac{1}{q(r'_{j})}}}\\ &=& \frac{1}{q(k)}\sum\limits_{\mathbf{r}\in R_{i\rightarrow k}^{t}}{\prod\limits_{j=1}^{t-1}{\frac{1}{q(r_{t-j})}}} \\ &=& \frac{1}{q(k)}\sum\limits_{\mathbf{r}\in R_{i\rightarrow k}^{t}}{\prod\limits_{m=1}^{t-1}{\frac{1}{q(r_{m})}}}, \end{array} $$

where the second equality is due to the equality **r** ^′^=(*r*1′,*r*2′,…,*r* *t*−1′)=(*r*_*t*−1_,*r*_*t*−2_,…,*r*_1_) resulting from correspondence between $R_{k\rightarrow i}^{Q^{t}}$ and $R_{i\rightarrow k}^{Q^{t}}$.

Thus, the Hastings ratio becomes

(11) $$ \frac{Q^{t}\left(i|k\right)}{Q^{t}\left(k|i\right)} = \frac{q(i)}{q(k)}.   $$

To guarantee that the Markov chain constructed with this proposal distribution has an equilibrium distribution (which is *P*(*G*|*D*)), we need to show that the chain is ergodic (i.e., irreducible and aperiodic). To prove the irreducibility of the chain, we first assume that *P*(*D*|*G*)>0 for all DAGs (as is the case when the hyperparameters are all positive and non-zero) and then show that each state is accessible from each other with transitions of arbitrary length. To see this, consider moving from a DAG *G* with *e* edges to a DAG *G* ^′^ with *e* ^′^ edges using *t*-transitions. This can be done with the following steps:

1. First, $d_{t} = \left \lfloor \frac {e}{t} \right \rfloor $ *t*-transitions are used to remove edges, where ⌊·⌋ is the floor function. The resulting graph has *e*−*t* *d*_*t*_ edges. Note that an edge can always be removed from an acyclic graph and the result is also acyclic.
2. Consider one more *t*-transition. Its first *e*−*t* *d*_*t*_−1=*d*_1_ steps are used to remove edges. The result is a graph with only one edge.
3. The next *t*−*d*_1_−1 steps of the *t*-transition are used to reverse the one remaining edge. The acyclicity is guaranteed for every graph with more than one node.
4. The last remaining step removes the only edge in the graph. The result is an empty graph.
5. Now note that if *G* ^′^ is a valid acyclic graph, it can be formed by adding its *e* ^′^ edges to an empty graph in any order and all the intermediate graphs will all be acyclic as well. For adding *e* ^′^ edges, $a = \left \lfloor \frac {e'}{t} \right \rfloor $ whole *t*-transitions and *e* ^′^−*a* *t* steps from one further *t*-transition are needed. To get rid of the extra *t*−(*e* ^′^−*a* *t*)=*E* steps in this partially needed transition, add any single edge of *G* ^′^ in the same direction as it appears in *G* ^′^ if *E* is even, or in the opposite direction if *E* is odd.
6. Make *E*−1 reversal operations on the only edge, the result being a graph with one edge in the opposite direction as in *G* ^′^.
7. Reverse the only edge and follow with *t*−*E*−1 additions of the edges in *G* ^′^. After this, the first *t*-transition is used.
8. Add the rest of the edges of *G* ^′^ with *a* *t*-transitions.

Aperiodicity of the chain can be proven with the same kind of proof as above. First, start by taking an integer *s* large enough so that the above kind of transition from *G* back to *G* via an empty graph can be made with *s* transitions of length *t*. The same kind of transition from *G* to *G* can also be made with *s*+1 transitions, since in addition to the exact same moves as in the first one with *s* transitions, it is possible to use the *t* moves of the one remaining transition in the empty graph by adding an edge, reversing it *t*−2 times and then deleting it. The period of the state is defined as $\gcd \left \{r:P_{t}^{(r)}\left (G_{i}|G_{i}\right)>0\right \}$, where $P_{t}^{(r)}\left (G_{i}|G_{j}\right)$ denotes the probability of going from model *G*_*j*_ to *G*_*i*_ with *r* steps of length *t* and $\gcd $ is a function returning the greatest common divisor. Because both *s* and *s*+1 belong to the set given as argument to function $\gcd $, the result is necessarily 1.

Note that due to the construction of the multi-step proposal distribution, a single DAG may be accessible via more than one walk, and thus, the probability of proposing it is also larger. It is also possible that a proposed transition of many steps actually leads to the starting structure or, e.g., just to a one-step neighbor in the structure space. Although these can be seen as inefficiency of the proposal distribution, the formation of such inefficient transitions is improbable as the number of structural neighbors can be quite large (simulation results not shown).

### Choosing the proposal distribution

The proposal can be easily modified for different datasets by tuning the transition lengths and the probabilities with which they are proposed. This tuning can be done adaptively, similarly as in several other areas where MCMC is used (e.g., [29]). Alternatively, the adaptive phase can be restricted to the burn-in phase to make sure the actual sampling is taken from the correct distribution.

A good method is to use mixtures of transitions with different lengths, i.e., to propose at each iteration a transition of length *t* with probability *p*_*t*_. We denote proposal distributions constructed in such a way with a vector [*p*_1_,*p*_2_,…,*p*_max_]. Note that the results derived in the previous section (Eqs. (10) and (11)) apply also here.

A loose upper bound for the highest usable jump size is 2 × (the maximal number of edges in a DAG) since with such a move, every structure can be reached from every other in one jump. This can be seen by considering that for a DAG with maximal number of edges, we can remove all of the edges and construct a new DAG by adding edges to the achieved empty graph. However, such jumps would not be sensible in practice as they are just randomly sampling the structure space and thus resulting in a very inefficient MCMC. Thus, in practical setting, the highest jump sizes should be limited to be much smaller than this loose upper bound.

On the other hand, the use of shorter jumps is sensible because with them, it is more efficient to explore the close-by DAGs which are likely to give a more representative sample of the current (local) maximum or drive the chain towards a near high-scoring DAG. Short jumps are also motivated by the fact that it is efficient to calculate the Bayes factor in the acceptance ratio if there are only a few changes to the structure due to canceling out of all the non-modified edges in the likelihood ratio.

We also note that some of the proposal distribution modifications presented earlier, e.g., edge reversal [11], optimal reinsertion operator [25], and inclusion order [24], can be seen as transitions capable of steps longer than one.

The computational cost of using a proposal distribution comprising a mixture of transitions of different lengths is close to using a proposal distribution with only one-step transitions. If we denote by *T*(*M*) the time complexity of making one move in the structure space and by *T*(*A*) the complexity of calculating the acceptance probability, then for a mixture proposal distribution, the time complexity is $T(A) + \sum _{i=1}^{t}{p_{i}\cdot i\cdot T(M)}$, where *p*_*i*_ is the probability of proposing a transition of length *i* as above. We can see that for rather small *t*, which are also the most usable for MCMC, the sum is close to *T*(*M*).

## Application to signaling network inference

To study the performance of the multi-step proposal distribution, we used the dataset from [5] which contains flow cytometry measurements of 11 proteins in a signaling network. The structure of this network is mostly known, but we aim at reconstructing it from the measurements only. The total number of utilized data points is 5400, containing both unstimulated (observational) cases and perturbations where some nodes have been either activated or inhibited. The data was preprocessed and discretized in the same way as in [5].

**Fig. 1:**
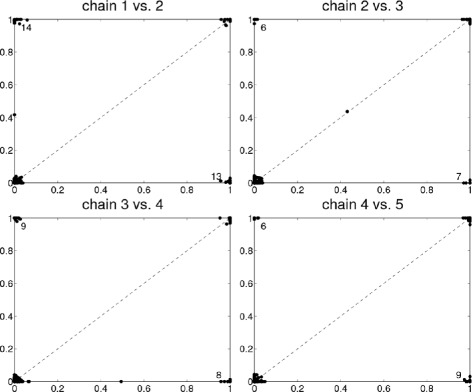
Edge posterior probabilities calculated from five different MCMC chains. The probabilities are compared to each other when the proposal distribution is the usual one-step version. Numbers in the *upper-left* and *lower-right* *corners*indicate the amount of overlapping dots, and in the titles, “*i* vs. *j*” denotes that edge posterior probabilities calculated from chain *i* are on the *x*-axis and those from chain *j* are on the *y*-axis. For visualization, a small amount of noise is added to the dots to better separate them from each other. The total number of dots (i.e., possible edges) is *N* ^2^=11^2^=121

We find that when trying to learn the structure of the signaling network as a BN in the normal setting using a proposal distribution with only one-edge modifications, the MCMC chains usually get stuck in local minima. This can be seen in Fig. 1 where edge posterior probabilities (6) from five different, independent chains are compared. The scatter plots show that the chains got stuck in different local maxima since if the samples were taken from the same area in the posterior, the estimated edge posterior probabilities should be similar, and thus, the points would lie close to the diagonal in the figure. Each of these chains was initiated with different random DAGs, and they were run for 900,000 steps (burn-in) and a sample of 100,000 DAGs was taken.

**Fig. 2:**
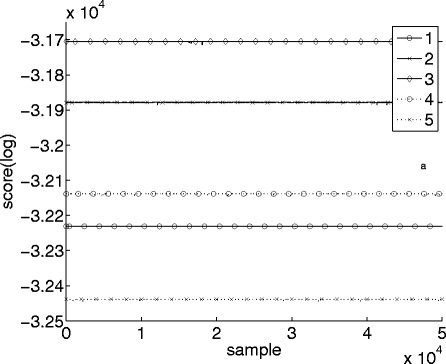
Scores calculated for the last 50,000 sampled graphs. The same five independent MCMC chains as in Fig. 1 were used. The proposal distribution was the standard one-step version

Convergence can also be investigated by plotting the scores of sampled DAGs from each chain, which is shown in Fig. 2 for the same five chains as above. The chains clearly do not reach the same area of search space but are all trapped in different local maxima, despite the long burn-in and sample periods.

**Fig. 3:**
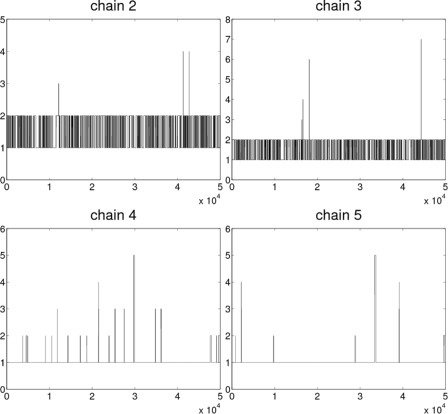
Trajectories of four MCMC chains in the sample phase. For each chain, the integers in the *y*-axis represent unique DAGs (which are in general not the same between chains despite being represented by the same integers). The last 50,000 sampled DAGs are shown

**Fig. 4:**
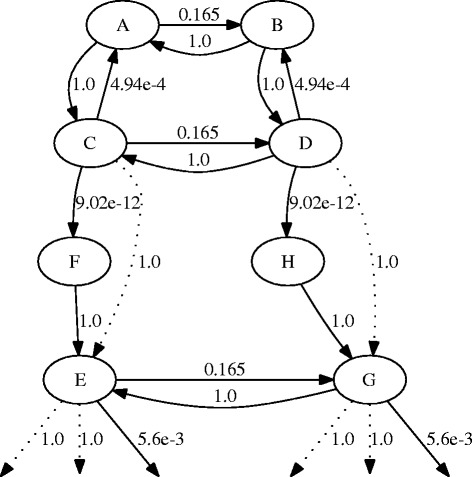
Local maximum of a chain. An illustration of a local maximum for one of the five chains in Figs. 1, 2, and 3 during its sample phase. The nodes A to H represent different DAGs. *Edges* denote the possible transitions of length 1 between the DAGs, with the *numbers* indicating the acceptance probabilities (i.e., the probability of accepting the move if it was proposed). Possible transitions of length 2 are shown with *dotted edges*. One of the local maxima in the case of the one-step proposal consists of DAGs C and D, between which the chain oscillates, since transitions elsewhere are very improbable. Only edges representing transitions with acceptance probability higher than 0.0001 are shown to make the picture more readable

When looking at the behavior of the example chains, it is noted that during the whole sample, the chains may practically remain in only one or two DAGs. This is illustrated in Fig. 3, where different DAGs visited by four of the example chains are shown. To see why this happens, Fig. 4 shows the situation for one of the chains. The chain is trapped in an area of local minimum where it just bounces between two different DAGs with relatively high acceptance probabilities. Transitions to all the rest of the neighboring DAGs are accepted with probabilities smaller than *p*=0.0001, meaning a rather improbable exit from this peak.

Results similar to the ones presented above occur frequently in trying to learn the BN structure. In fact, finding chains converged to the same area in DAG space seems to be a special case with this data.

**Fig. 5:**
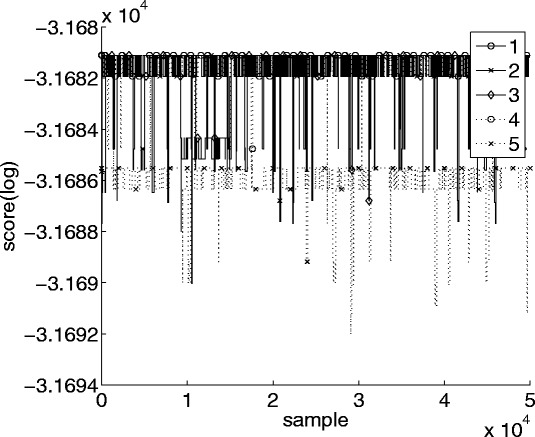
DAG scores when the used proposal distribution was [*p*_1_=0.5,*p*_2_=0.5]. Scores were calculated for samples of 50,000 DAGs from the ends of five independent MCMC chains

Next we tested with a proposal distribution that proposes both steps of lengths 1 and 2 with equal probabilities, i.e., [ *p*_1_=0.5,*p*_2_=0.5]. Figure 5 shows the score plots again for five chains with this proposal distribution. One chain (number 5) seems to be stuck at a more lower scoring area than the four others, although its score is very close to the top ones.

**Fig. 6:**
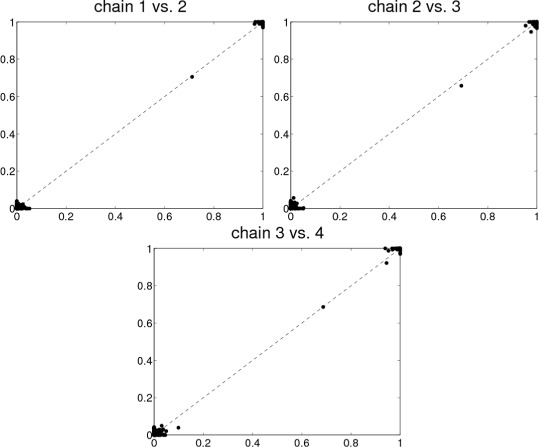
Edge posterior probabilities when the proposal distribution proposes two steps with probability 0.5 and normal one step with probability 0.5. Edge posterior probabilities were calculated from four different MCMC chains (chains 1 to 4 in Fig. 5) and compared to each other

Excluding chain 5, the scatter plots in Fig. 6 confirm that the four remaining chains have converged to the same area and represent the same posterior distribution.

Allowing transitions of length 2 makes it possible for the chains to escape local maxima easier. As an example of this, consider the same local maximum area as shown in Fig. 4. When the chain is allowed to make transitions of length 2 (indicated by the dotted edges), a way out of the local maximum is introduced. Thus, it can be seen that in this case, the practically inaccessible DAGs (as can be seen from transition probabilities of C →F and D →H) can be jumped over with transitions of length 2. Note that these longer proposal moves make available not only high-scoring network structures E and G but also their neighbors (see outgoing edges from nodes E and G). Depending on the situation, convergence might be further accelerated by using even longer possible transitions.

**Fig. 7:**
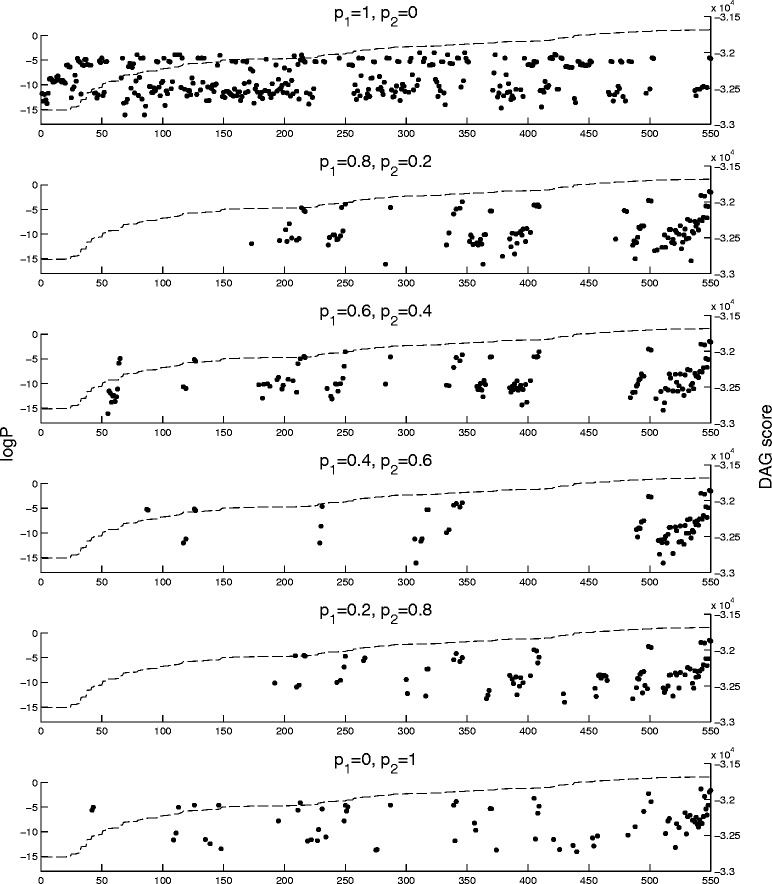
Sampled posteriors using different proposal distributions. For each different one-step and two-step combination shown in the figure, 100 chains were run with random initial DAGs for a burn-in period of 1,000,000 and a sample of 100,000 was taken. Each position on the *x*-axis represents a different DAG, sorted by ascending score, which is shown with a *dashed line*. The *x*-axes are the same between subfigures so a DAG at position *k* is the same in all subfigures. The posterior probabilities of each DAG in the samples are shown with *dots* (zero values not shown)

To further test how different proportions of one and two steps affect convergence and sampling characteristics, we selected six different proposal distributions having varying proportions of one and two steps ([*p*_1_=*p*,*p*_2_=1−*p*]for *p*=0,0.2,0.4,0.6,0.8,1). For each of these, 100 chains with random start points were ran each for 1,000,000 steps followed by taking a sample of 100,000 DAGs. For each proposal distribution, the samples were then combined, yielding samples of size 10,000,000, to obtain a view of how the posterior was sampled, as seen in Fig. 7.

Again, the difference in convergence is evident between proposals having two steps and the one without them. The one-step proposal chains sample largely areas of lower scoring DAGs and are mostly unable to find the highest scoring DAGs. Importantly, we observe that there is no single DAG standing out as the best scoring structure; instead, there are tens of DAGs with about equal scores.

Figure 7 demonstrates that the probability of reaching these highest scoring DAGs is remarkably higher when using more versatile proposal distributions than when relying only on the standard one-step version. This is seen, for example, in the lack of DAGs with an order number greater than 500 in one-step proposal runs, as well as in that the posterior probabilities for the highest scoring DAG are about 400 times larger for the tests where also two steps are used in the proposal distributions.

To characterize the sampling of DAG space with different proposal distributions, we used the same runs as in Fig. 7 and calculated the numbers of DAGs visited during the sampling period of each chain and took the mean of those numbers over the six different proposal distributions (Table 1). Looking at the numbers reveals that longer steps allow more efficient sampling in terms of sampled DAGs, as they are able to propose transitions to a much larger neighborhood and step over low-scoring DAGs. Notable is also the low number of visited DAGs in the proposal using only two steps, which results from the proposal being unable to propose many DAGs just one modification away, which may contain DAGs having comparable scores or belonging to the same equivalence class. Thus, while steps of length 2 increase probability of convergence as shown above, including also steps of length 1 gives a more “versatile” sample in terms of numbers of sampled DAGs.

**Table 1:** Mean and standard deviation of numbers of DAGs visited during sampling periods of the same chains as in Fig. 7

Proposal distribution	Mean DAGs (std)
*p*_1_=1.0,*p*_2_=0.0	4.41 (3.3698)
*p*_1_=0.8,*p*_2_=0.2	8.3 (2.8373)
*p*_1_=0.6,*p*_2_=0.4	8.01 (2.4058)
*p*_1_=0.4,*p*_2_=0.6	7.24 (3.0021)
*p*_1_=0.2,*p*_2_=0.8	6.31 (3.212)
*p*_1_=0.0,*p*_2_=1.0	2.66 (2.4994)

**Fig. 8:**
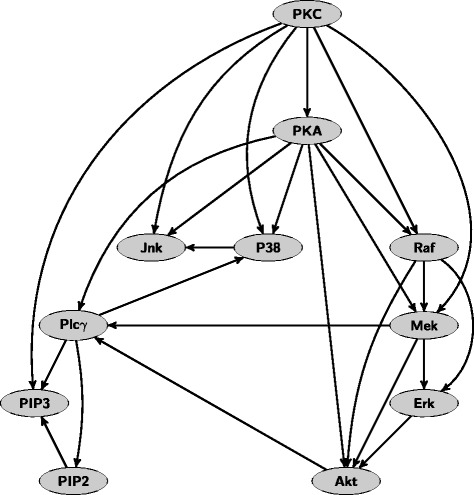
MAP DAG from the combined sample in Fig. 7 when the used proposal distribution was [*p*_1_=0.8,*p*_2_=0.2]

Figure 8 shows the maximum a posteriori (MAP) DAG obtained from the combined samples of chains where the proposal distribution was [*p*_1_=0.8,*p*_2_=0.2]. Compared to the one shown in [5], this graph scores considerably better (−3.4367·10^4^ vs. −3.1682·10^4^) and so does the average graph (with edges present if posterior of edge >0.85, score −3.2460·10^4^). Most of the edges in the MAP DAG are the same as in [5], but there are a couple of differences. Perhaps the most notable difference is the number of edges between the nodes of the triad Plc *γ*, PIP3, and PIP2. This triad is separated from the rest of the network in the result of [5] even though it is known to have connections. Our network captures some of these, though it highlights the need for further interventional measurements to be made to this triad in order to learn the correct causal relationships.

**Fig. 9:**
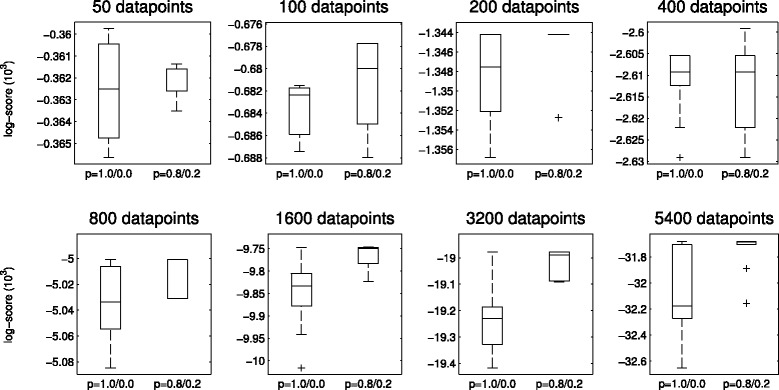
Scores of MAP DAGs for different dataset sizes. Scores were calculated with the normal one-step proposal distribution (i.e., [*p*_1_=1.0, *p*_2_=0.0], marked *p*=1.0/0.0) and a proposal distribution with also steps of length 2 ([*p*_1_=0.8,*p*_2_=0.2], marked *p*=0.8/0.2). For each chain, the burn-in was 1,000,000 and the sample size was 50,000. The scores were calculated 10 times, using different sampled datasets

***Effect of dataset size*** A growing dataset size should help identify the true underlying DAG, but it can also render the posterior more peaky, making it harder for the MCMC chains to traverse it. To see how the performance varies with varying dataset size, we sampled different numbers of data points from the whole dataset and compared the MAP results of two different proposal distributions (see Fig. 9). As can be seen, with smaller datasets, the one-step proposal works fine, but for any of the larger ones, the chains get stuck in lower score areas of the posterior landscape, while when allowing longer (here two steps) jumps, the chains are able to escape these.

## Comparison using simulated datasets

**Fig. 10:**
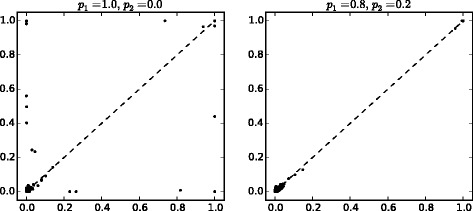
Edge posterior probabilities for simulated data from the Alarm network. Edge posterior probabilities for the best pairs of chains among four different MCMC chains, when using two different proposal distributions

To test the effect of using steps larger than one in the case of simulated datasets, we utilized the well-known Alarm network [30]. We compared the performance of MCMC using two different proposal distributions, one with [*p*_1_=1.0] and another with [*p*_1_=0.8,*p*_2_=0.2], using the Alarm network to generate datasets containing 500 measurements of which 30 % were interventions. For both proposal distributions, we ran four independent MCMC chains using random initial DAGs, burn-in of 500,000 and sample of 1,000,000 out of which every 1000th DAG was taken. A maximum fan-in of 5 was used in all the simulations. For both proposal distributions, we identified the pairs of chains that showed the best convergence by choosing the chain pair with the lowest sum of squared differences between their edge posterior probability estimates, and the scatter plots are shown for these in Fig. 10. The minimum sums of squared differences were 21.936 for [*p*_1_=1.0] and 0.0102 for [*p*_1_=0.8,*p*_2_=0.2].

In many practical settings, the modeled systems contain loops and hidden variables and the structure of such a system is not thus perfectly modeled as an acyclic graph. To mimic this situation with simulated data, we took an approach where we generated data from two different random BNs, then combined them to one dataset and tried to learn the structure from the combined dataset. We used networks with 10 nodes, generated 3000 data points from both random BNs (totaling 6000 data points), and then ran for both [*p*_1_=1.0] and [*p*_1_=0.8,*p*_2_=0.2] three MCMC chains each with different initial DAGs. For both of these proposal distributions, we calculated as a measure for convergence the sums of squared differences in estimated edge posterior probabilities between each possible pair in the set of these three chains. This was done six times, and the average was calculated over the sums of squared differences, yielding 0.352 for [*p*_1_=1.0] and 0.223 for [*p*_1_=0.8,*p*_2_=0.2]. The differences are not big, but there seem to be benefits in using proposal distributions with larger than one step also in this scenario.

## Conclusions

For many types of data, the posterior probability over models can be highly multimodal, and thus, there is no single model or equivalence class standing out. This is at least partly due to the fact that the data was generated by a system not perfectly representable as a Bayesian network, although the effect is also to some extent present in simulated datasets. Regardless of the sought-after posterior being over models or features, it is important in such cases that the sample from the posterior covers the greater part of these high-scoring structures in order for the sample to be representative enough. One possible method is to start several MCMC chains with different start points and combine the samples from these, as otherwise the needed sampling from one chain might be excessively large. In this case, it is still advisable to use an efficient proposal distribution to prevent the chains from being stuck in low-scoring local maxima and producing a less representative sample.

The advantage of using the presented multi-step proposal distribution over the other constructs, such as the inclusion boundary methods or others, is its simplicity and low demand for computation. The proposal distribution is not guaranteed to outperform all existing proposal distribution variants in every scenario, but it is general and flexible in the sense that it can be easily tuned, by varying transition lengths and varying mixes of different transition lengths, unlike other constructs. Therefore, it allows an easy implementation of adaptable MCMC for Bayesian network structure learning, where the proposal is tuned during the burn-in period, or provides a framework for the development of full adaptive samplers. Importantly, unlike other recently proposed structure inference methods which make use of e.g. node ordering or dynamic programming, the multi-step proposal distribution proposed here allows a straightforward use of informative priors of structures typically present in biological applications.
